# The Role of Rv1476 in Regulating Stress Response and Intracellular Survival of *Mycobacterium tuberculosis*

**DOI:** 10.3390/cimb46020100

**Published:** 2024-02-16

**Authors:** Aikebaier Reheman, Yifan Wang, Huaiyuan Cai, Pingyang Wei, Gang Cao, Xi Chen

**Affiliations:** 1 National Key Laboratory of Agricultural Microbiology, College of Veterinary Medicine, Huazhong Agricultural University, Wuhan 430070, China; akr0902@webmail.hzau.edu.cn (A.R.); giggleral@163.com (Y.W.); wikk1019@163.com (H.C.); a895588594@gmail.com (P.W.); 2 Bio-Medical Center, Huazhong Agricultural University, Wuhan 430070, China

**Keywords:** *Mycobacterium tuberculosis*, intracellular survival, *Rv1476*, resistance, macrophages

## Abstract

The virulence of *Mycobacterium tuberculosis* (*M. tuberculosis*) is related to many factors, including intracellular survival, cell wall permeability, and cell envelope proteins. However, the biological function of the *M. tuberculosis* membrane protein *Rv1476* remains unclear. To investigate the potential role played by *Rv1476*, we constructed an *Rv1476* overexpression strain and found that overexpression of *Rv1476* enhanced the intracellular survival of *M. tuberculosis*, while having no impact on the growth rate in vitro. Stress experiments demonstrated that the *Rv1476* overexpression strain displayed increased susceptibility to different stresses compared to the wild-type strain. Transcriptome analysis showed that *Rv1476* overexpression causes changes in the transcriptome of THP-1 cells, and differential genes are mainly enriched in cell proliferation, fatty acid degradation, cytokine–cytokine receptor interaction, and immune response pathways. *Rv1476* overexpression inhibited the expression of some anti-tuberculosis-related genes, such as *CCL1*, *IL15*, *IL16*, *ISG15*, *GBP5*, *IL23*, *ATG2A*, *IFNβ*, and *CSF3*. Altogether, we conclude that *Rv1476* may play a critical role for *M. tuberculosis* in macrophage survival.

## 1. Introduction

Tuberculosis (TB) is a chronic infectious disease caused by *Mycobacterium tuberculosis* (*M. tuberculosis*) [1]. According to the World Health Organization (WHO), there were 5.8 million new cases of TB and 1.5 million TB-related deaths in 2021 [2]. However, the long-term latency and drug resistance of *M. tuberculosis* has not yet been fully resolved. *M. tuberculosis* establishes a dynamic equilibrium with the host immune system, which persists throughout life without any noticeable signs or symptoms of disease [3]. About 10% people will develop active TB, especially within a few years of exposure [4]. Although active TB can be overcome with long-term use of anti-TB drugs, the rise of drug-resistant TB poses a major challenge to global TB control. Therefore, an in-depth study of the function of *M. tuberculosis* genes will play an important role in revealing the pathogenic mechanism of *M. tuberculosis* and exploring potential anti-TB drug targets. 

Macrophages are intracellular bacterial scavengers and key immune cells in the innate immune response against *M. tuberculosis*. After entering the lungs, *M. tuberculosis* is phagocytosed by alveolar macrophages [5,6]. As the first line of defense in the host’s innate immune system, macrophages eliminate intracellular pathogens through a range of mechanisms, including phagolysosomal fusion, cytokine secretion, apoptosis, and autophagy [7,8,9]. *M. tuberculosis* can evade the immune system of the host by utilizing its distinct immune mechanism [10,11,12]. Additionally, *M. tuberculosis* can acquire carbon and energy within the macrophages, allowing it to sustain its survival in the host body for an extended period [13]. Therefore, a deeper understanding of the interaction between *M. tuberculosis* and macrophages will help to unravel the immune evasion mechanism of *M. tuberculosis*. 

Early proteomics study has showed Rv1476 is associated with *M. tuberculosis* cell membrane components [14,15], and *Rv1476* encodes a membrane protein that was identified in membrane protein fractions and whole-cell lysates of *M. tuberculosis* [16]. Minato et al. constructed a transposon mutation library to screen essential genes for *M. tuberculosis*, and the results showed that *Rv1476* is an essential gene for *M. tuberculosis* [17]. However, the biological function of *Rv1476* has not yet been characterized. Previously, we developed an *M. tuberculosis* endogenous type III-A CRISPR/Cas10 system to conduct a screening of intracellular survival-related genes. The results revealed a significant reduction in the survival rate of *M. tuberculosis* in macrophages following *Rv1476* knockdown [18]. In the current study, we overexpressed *Rv1476* in *M. tuberculosis* and found the *Rv1476* overexpression strain was more susceptible to different stresses but was beneficial to survival within macrophages. Therefore, we speculate that *Rv1476* may be related to *M. tuberculosis* intracellular survival. 

## 2. Materials and Methods

### 2.1. Bacterial Strains and Cell Culture

*M. tuberculosis* H37Ra was cultured in Middlebrook 7H9 broth (Becton, Dickinson, Franklin Lakes, NJ, USA) supplemented with 0.5% glycerol, 0.05% Tween 80, and 10% oleic acid albumin dextrose catalase (OADC, Becton, Dickinson). Escherichia coli DH5α for DNA cloning was grown in Luria–Bertani medium. A weight of 50 μg/mL kanamycin was added as needed. 

Human monocytes (THP1, ATCC TIB-202) were cultured in RPMI 1640 medium (Gibco, Grand Island, NY, USA) containing 10% FBS at 37 °C, 5% CO_2_.

### 2.2. Construction of Rv1476 Overexpression Strains

To construct the *Rv1476* overexpression *M. tuberculosis* strain, the *Rv1476* coding region was amplified by using specific primers (Table 1) and inserted into vector pMV261 to obtain a pMV-Rv1476 plasmid. The recombinant plasmid pMV-Rv1476 was electrotransformed into the *M. tuberculosis* H37Ra strain, and overexpression strains were screened under kanamycin-resistant conditions. The overexpression strain was confirmed by qPCR using specific primers (Table 1), and was named M. tb-Rv1476, and the strain transformed with the empty vector was used as a control and named with M. tb-pMV261.

### 2.3. Survival Analysis under Different Stress Conditions

To detect the survival of M. tb-pMV261 and M. tb-Rv1476 under different stress conditions, the M. tb-pMV261 and M. tb-Rv1476 were cultured with 7H9 until the OD_600nm_ reached 0.8. For surface stress, bacteria were treated with 0.05% SDS for 0 h, 1 h, 2 h, and 3 h. To test sensitivity to lysozyme, bacteria were treated with 2.5 mg/mL lysozyme for 0 h, 3 h, and 6 h. For peroxide stress, bacteria were treated with 6 mM H_2_O_2_ for 0 h, 2 h, 4 h, and 8 h. At each time point, 200 μL samples were collected and bacterial activity was measured using the Alamar blue assay.

### 2.4. In Vitro Growth and Intracellular Survival

For growth curve assay, the M. tb-pMV261 and M. tb-Rv1476 were cultured with 7H9 until the OD_600nm_ reached 0.8. The bacteria was inoculated into the new culture medium and its OD_600nm_ adjusted to 0.001. The OD_600nm_ was detected and analyzed at indicated time points. 

For intracellular survival assay, 2.0 × 10^5^ THP-1 cells were seeded in a 24-well plate and differentiated with PMA for 24 h, the THP-1 cells were incubated with M. tb-pMV261 and M. tb-Rv1476 (MOI = 10) for 4 h. To stop infection, the cells were washed three times with pre-warmed PBS to remove extracellular bacteria and then supplied with a fresh medium with 5% FBS containing amikacin (50 mg/mL) (referred to as day 0). The cells were lysed after 0 h, 48 h, and 96 h infection using sterile 0.1% Tween-80 in water, and viable bacilli were enumerated by serial dilution of lysates and plating on Middlebrook 7H11 agar. CFUs were counted after 3 to 4 weeks of culture. All the infections were performed in triplicate.

### 2.5. Host Cell Transcriptome Analysis

A number of 2.5 × 10^6^ cells were seeded in a 6-well plate and differentiated with PMA for 24 h, and the THP-1 cells were incubated with M. tb-pMV261 and M. tb-Rv1476 (MOI = 10) for 4 h. To stop infection, the cells were washed three times with pre-warmed PBS to remove extracellular bacteria and then supplied with a fresh medium with 5% FBS containing amikacin (50 mg/mL) (referred to as day 0). The cells were lysed with 1 mL of TRIzol reagent after 72 h infection. We added 200 μL of chloroform to the lysate and centrifuged it at 12,000× *g* for 10 min at 4 °C. We then transferred the supernatant to a new 1.5 mL RNase-free tube and added an equal volume of pre-cooled isopropanol. To better precipitate RNA, the mixture was maintained at −20 °C overnight, and then pelleted by centrifuge f at 12,000× *g* for 10 min at 4 °C; the precipitated RNA was then washed twice with 75% ethanol. The concentration and quality of total RNAs were detected by a Nanodrop 2000c spectrophotometer (Thermo Scientific, Waltham, MA, USA). 

The libraries were generated with VAHTS Universal V6 RNA-seq Library Prep Kit for MGI (Vazyme, NRM604). Briefly, mRNAs were isolated using mRNA Capture Beads from 1μg of total RNA and incubated for fragmentation at 94 °C for 8 min to obtain mRNAs with a length of 150–200 bp. The mRNAs were reverse-transcribed to cDNA and converted into double-stranded cDNA molecules. Following end-repairing and dA-tailing, the pair-ended sequencing adaptors were ligated to the ends of the cDNA fragments and then subjected to library amplification and purification. The prepared library was sequenced on the MGISEQ-2000 (BGI, Shenzhen, China) platform.

### 2.6. qRT-PCR

The total RNAs were extracted from cells using RNAiso Plus reagent (Code No. 9109, TaKaRa, Shiga, Japan), and then 2 μg of RNAs were reverse-transcribed to cDNA using ABScript III RT Master Mix (RK20428, ABclonal, Wuhan, China) according to the manufacturer’s instruction. Briefly, we added 4 μL 5 × ABScript III RT Mix to 2 μg total RNA and mixed it thoroughly, then added nuclease-free water to make it 20 μL and carried out the reverse transcription reaction according to the following reaction procedure (55 °C, 15 min, 85 °C, 5 min, 4 °C, hold). Gene expression was analyzed by qRT-PCR using SYBR Select Master Mix (T11225, TOYOBO, Osaka, Japan). All of the reactions were triplicated and performed in the QuanStudio 6 Real-time PCR system. Further, the Ct values for each gene amplification were normalized with internal control GAPDH by the 2^−ΔΔCt^ methods. The qRT-PCR primers are shown in Table 1.

### 2.7. RNA-Seq Data Analysis

The RNA-seq data was processed into a reading count corresponding to each sample following the method of Pertea et al. [19], and then read count lists were merged into a matrix by an R package, dpylr 1.1.4 (https://CRAN.Rproject.org/package=dplyr) accessed on 10 October 2023. The read count matrix was applied to DESeq2 [20]. For the differential analysis, genes with abs (foldchange) > 2 and *p* value < 0.05 were considered as the significant up/down-regulation genes; meanwhile, the normalized count was also exported. The volcano plot was generated by R package ggplot2 3.4.4 (https://ggplot2.tidyverse.org) accessed on 14 October 2023 and ggrepel 0.9.4 (https://CRAN.R-project.org/package=ggrepel accessed on 14 October 2023). The functional enrichment was performed using the R package clusterProfiler [21]. A heatmap was generated by the R package pheatmap 1.0.12 (https://CRAN.R-project.org/package=pheatmap accessed on 25 October 2023).

### 2.8. Statistical Analysis

Numerical data were analyzed by GraphPad Prism 7.0 (La Jolla, CA, USA) software from three independent experiments, shown as mean ± SEM. Evaluation of the significance of differences between groups was performed by using one-way ANOVA or Student’s *t*-test. Statistical difference was considered to be significant when *p* < 0.05. * *p* < 0.05, ** *p* < 0.01, *** *p* < 0.001, and **** *p* < 0.0001. All experiments were performed in triplicate.

## 3. Results

### 3.1. Rv1476 Reduces the Stress Resistance of M. tuberculosis

**Figure 1:**
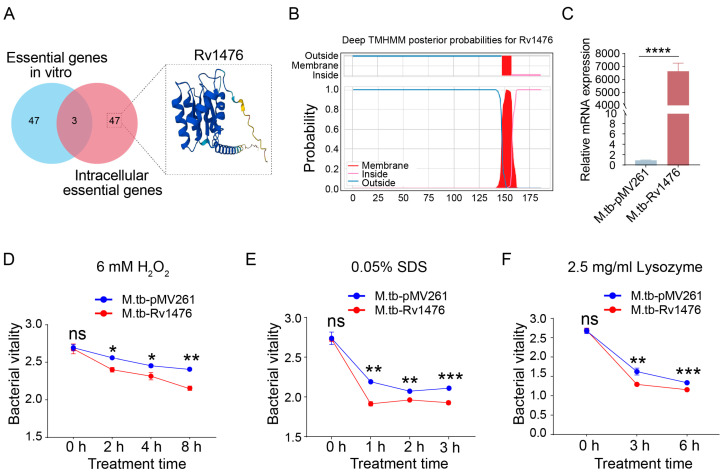
*Rv1476* attenuates the growth viability of *M. tuberculosis* under various stress conditions. (**A**) Screening of *M. tuberculosis* gene *Rv1476* using CRISPRi method; the protein 3D structure was generated by AlphaFold. (**B**) TMHMM server showed Rv1476 protein has one transmembrane domain. (**C**) Detection of *Rv1476* mRNA expression using qPCR. (**D**–**F**) M. tb-pMV261 and M. tb-Rv1476 growth in 7H9 media containing 6 mM H_2_O_2_, 0.05% SDS and 2.5 mg/mL Lysozyme, respectively, at indicated times. At each time point, the survival was determined by Alamar blue assay. * *p* < 0.05, ** *p* < 0.01, *** *p* < 0.001, and **** *p* < 0.0001.

The results showed that *Rv1476* might be an essential gene for *M. tuberculosis* survival in macrophages but not in vitro (Figure 1A). The TMHMM results showed that Rv1476 contains one transmembrane domain at aa 148–157 (Figure 1B). Herein, we constructed an *Rv1476* overexpression *M. tuberculosis* strain (Figure 1C), and tested the stress-resistance ability of the M. tb-Rv1476. To assess the impact of *Rv1476* on reactive oxygen stress, M. tb-pMV261 and M. tb-Rv1476 were cultured with 7H9 containing 6 mM H_2_O_2_ for different time points. The results revealed that the growth vitality of M. tb-Rv1476 was notably lower compared to that of M. tb-pMV261 (Figure 1D). Similar results were found when M. tb-pMV261 and M. tb-Rv1476 were cultured with 7H9 containing 0.05% SDS and 2.5 mg/mL lysozyme for different time points (Figure 1E,F). These founds suggested that overexpression of *Rv1476* reduces the stress resistance of *M. tuberculosis*.

### 3.2. Rv1476 Potentiates M. tuberculosis Survival in Macrophage

**Figure 2:**
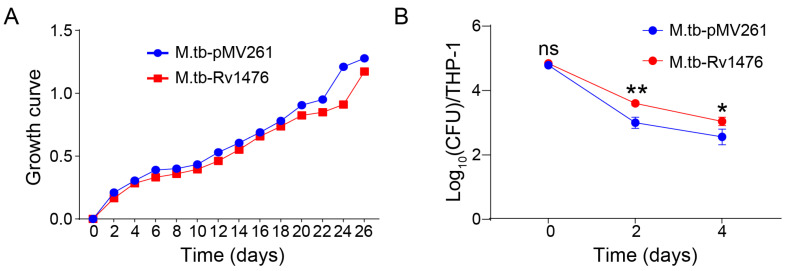
*Rv1476* improved the intracellular survival of *M. tuberculosis*. (**A**) M. tb-pMV261 and M. tb-Rv1476 growth curve in 7H9 media containing 10% OADC. (**B**) The intracellular survival rate of M. tb-pMV261 and M. tb-Rv1476. ns -not significant, * *p* < 0.05 and ** *p* < 0.01.

Growth curve testing showed that there was no significant difference between the growth rates of M. tb-Rv1476 and M. tb-pMV261 (Figure 2A). The results indicating that the influence of *Rv1476* on the in vitro growth of *M. tuberculosis* are negligible. Next, we performed a survival assay and found that overexpression of *Rv1476* significantly increased *M. tuberculosis* survival in macrophages compared to M. tb-pMV261 (Figure 2B). This suggested that overexpression of *Rv1476* can improve the survival rate of *M. tuberculosis* in macrophages.

### 3.3. Rv1476c Regulates Macrophage Gene Expression Profile

**Figure 3:**
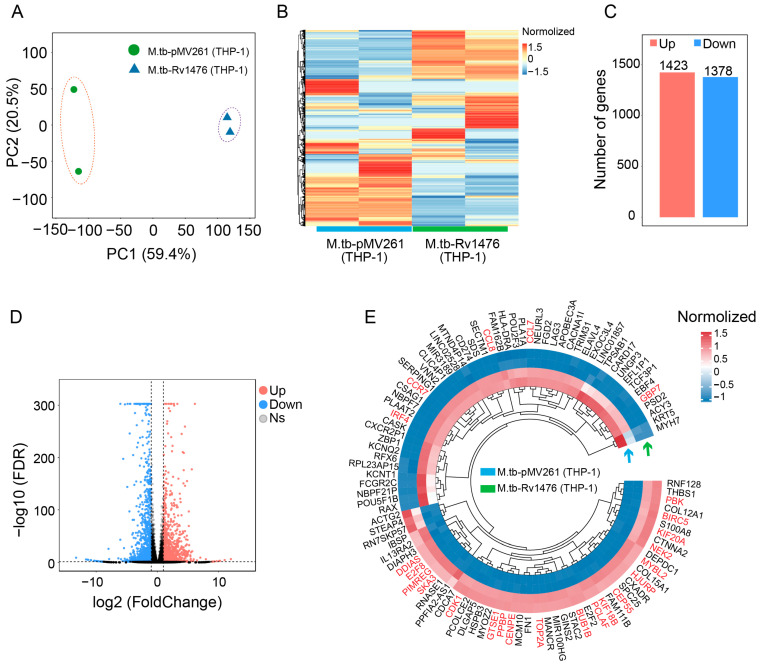
Detection and analysis of DEGs between M. tb-pMV261- and M. tb-Rv1476-infected THP-1 cells. (**A**) Principal component analysis (PCA) of transcriptomic variations. (**B**) The heat map represents the expression of all genes; red indicates high, and blue indicates low expression levels. (**C**) The number of all differential genes, 1423 up-regulated genes and 1378 down-regulated genes. (**D**) Volcano plot represents differentially expressed genes. Red dots represent significantly up-regulated genes, blue dots represent significantly down-regulated genes, and gray dots represent genes with no significant difference. (**E**) The circular heat map represents the top 100 differentially expressed genes, with red indicating up-regulated genes and blue indicating down-regulated genes.

To further understand the mechanism of *Rv1476* in promoting the intracellular survival of *M. tuberculosis*, we analyzed the gene expression profile of macrophages infected with M. tb-Rv1476 by using RNA-seq. Our results showed that overexpression of *Rv1476* caused a comprehensive differential gene expression of macrophages compared to M. tb-pMV261 (Figure 3A,B). Among these differential genes, 1423 genes are up-regulated and 1378 genes are down-regulated (Figure 3C,D). The top 100 differential genes showed that genes associated with cell division and cell cycle pathways were up-regulated, whereas genes related to immune cell migration and chemotaxis pathways were down-regulated (Figure 3E).

### 3.4. Pathway Enrichment Analysis of Differentially Expressed Genes

**Figure 4:**
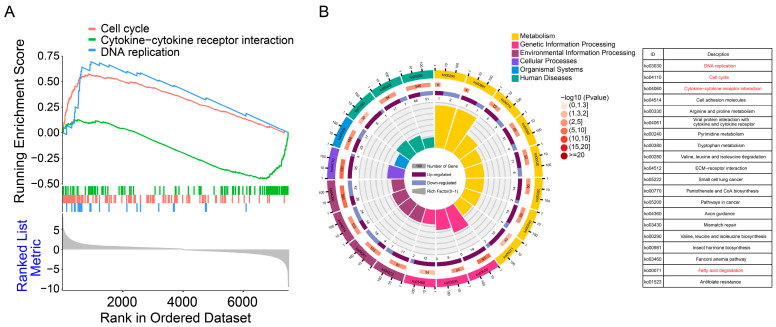
Pathway analysis of all differential genes. (**A**) GSEA analysis of the transcriptome. A pathway of positive enrichment score is up-regulated, whereas a pathway of negative enrichment score is down-regulated. (**B**) The enrichment circle plot was used to display the enrichment analysis of all differential genes.

To better understand the functions of differentially expressed genes, functional enrichment analysis was performed on differentially expressed genes. GSEA analysis results indicate that the differentially expressed genes are predominantly enriched in pathways related to cell cycle, cytokine–cytokine receptor interaction, and DNA replication pathways (Figure 4A). KEGG analysis showed 20 pathways; the top 3 pathways were DNA replication, cell cycle, and cytokine–cytokine receptor interaction pathways (Figure 4B). Notably, certain differential genes exhibited enrichment in the fatty acid degradation pathway, which is closely associated with the intracellular survival of *M. tuberculosis*. 

### 3.5. Rv1476 Affects the Expression of Anti-Tuberculosis-Related Genes

**Figure 5:**
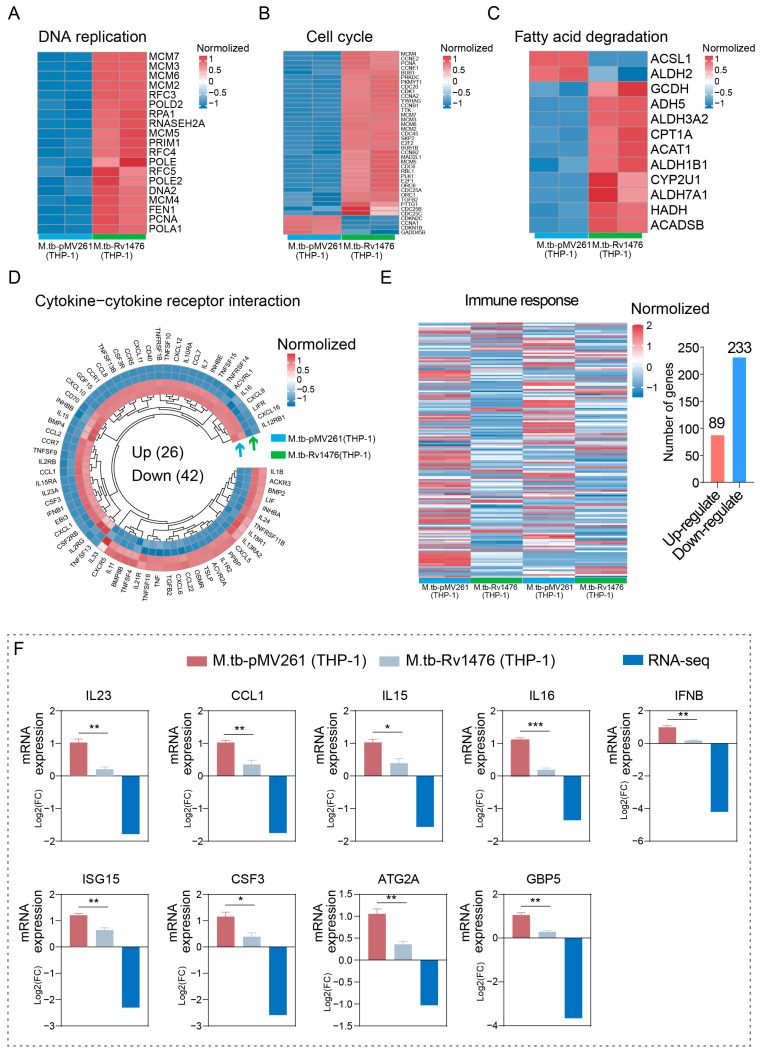
Displayed differentially expressed genes enriched in key pathways. (**A**) Differentially expressed genes in DNA replication pathways. (**B**) Differentially expressed genes in cell cycle pathways. (**C**) Differentially expressed genes in fatty acid degradation pathways. (**D**) Differentially expressed genes in cytokine–cytokine receptor interaction pathways. (**E**) Differentially expressed genes in immune response pathways. (**F**) The gene expression level was validated by qPCR. * *p* < 0.05, ** *p* < 0.01 and *** *p* < 0.001.

To better understand the impact of *Rv1476* on the gene expression profile of host cells, we further displayed the expression levels of genes related to DNA replication, cell cycle, fatty acid degradation, cytokine–cytokine receptor interaction, and immune response. We found that genes associated with the DNA replication pathway exhibit up-regulation (Figure 5A). The majority of genes enriched in the cell cycle and fatty acid degradation pathways display up-regulation (Figure 5B,C). Functional analysis results showed that 68 differentially expressed genes were enriched in the cytokine–cytokine receptor interaction pathway, of which 26 were up-regulated and 42 were down-regulated (Figure 5D). It is worth noting that 322 differentially expressed genes were enriched in the immune response pathway, of which 89 genes were up-regulated and 233 genes were down-regulated (Figure 5E). 

Subsequently, we further verified the transcription levels of genes on anti-tuberculosis-related genes such as *CCL1*, *IL15*, *IL16*, *ISG15*, *GBP5*, *IL23*, *ATG2A*, *IFNβ*, and *CSF3*. The results showed that all the genes expression were down-regulated in THP-1 cells infected with M. tb-Rv1476 compared with M. tb-pMV261 (Figure 5F). 

## 4. Discussion

The innate immune system plays a crucial role in the defense against *M. tuberculosis* infection. Macrophages, as key innate immune cells, employ different cellular processes like phagocytosis, autophagy, apoptosis, and inflammasome formation to combat invading *M. tuberculosis* [22,23]. However, *M. tuberculosis* resists macrophage clearance through some strategies to ensure its persistence within macrophages, such as inhibiting autophagy, inflammatory response, and apoptosis [11,24]. Therefore, a better understanding of the interaction mechanism between *M. tuberculosis* and the immune system is of vital significance for the treatment and prevention of TB. 

The cell wall of *M. tuberculosis* is rich in various lipids, which are thought to play an important role in resisting host immune clearance [25,26,27]. Previously, we used whole-genome CRISPRi screening and found that Rv1476 is beneficial to the intracellular survival of *M. tuberculosis* [18]. Previous to this, a proteomic study showed that Rv1476 is predicted to be a transmembrane protein [14], and our TMHMM results showed that Rv1476 contains one transmembrane domain at aa 148–157. Transmembrane proteins play an important role in intracellular signal transmission, nutrient absorption, and resistance to stressful environments. However, there are currently few reports on the biological functions of *Rv1476*. In the current study, we found that overexpression of *Rv1476* promoted *M. tuberculosis* intracellular survival in host macrophages. Interestingly, overexpression of *Rv1476* makes *M. tuberculosis* more susceptible to stress environments and reduces the proliferation activity of *M. tuberculosis*, which may be due to the increase in cell wall permeability. This result is consistent with a report by Ruan et al. [28]. 

As an intracellular bacterium, *M. tuberculosis* mainly lives in macrophages, which regulates its intracellular survival by changing the gene expression profile of macrophages, especially the expression of anti-tuberculosis immune-related genes [29,30,31]. Exploring the regulation of macrophage gene expression profiles by *M. tuberculosis* genes is of great significance for understanding the long-term survival of pathogens in host cells. In the current study, the *Rv1476* overexpression strain caused changes in the transcriptome of THP-1 cells. The results showed that genes related to immune cell migration and chemotaxis were down-regulated. These results suggest that *Rv1476* might function by affecting immune response-related genes. Further, we conducted pathway enrichment analysis on the differentially expressed genes and observed that these genes were primarily enriched in pathways associated with cell proliferation, fatty acid degradation, cytokine–cytokine receptor interaction, and immune response. The majority of genes enriched in the cell proliferation and fatty acid degradation pathways show up-regulation, whereas the majority of genes enriched in the cytokine–cytokine receptor interaction and immune response pathways exhibit down-regulation. It is suggested that *Rv1476* may affect the expression of genes related to cell proliferation and immune response pathway. Apoptosis is a crucial mechanism for macrophages to eliminate intracellular *M. tuberculosis* [32]. However, *M. tuberculosis* employs various mechanisms to hinder macrophage apoptosis, thus evading its destruction by the macrophages [33,34]. Host-derived fatty acids are an important carbon source during *M. tuberculosis* infection and facilitate the growth of *M. tuberculosis* [35]. Based on our results, it is likely that *Rv1476* inhibits macrophage apoptosis and regulates the up-regulation of genes associated with cell proliferation and fatty acid degradation. Additionally, it may also down-regulate genes related to cytokines and immune response, thereby preventing its clearance by macrophages. The expression levels of certain genes related to anti-tuberculosis were further confirmed, such as *CCL1*, *IL15*, *IL16*, *ISG15*, *GBP5*, *IL23*, *ATG2A*, *IFNβ*, and *CSF3*. The results demonstrated that *Rv1476* led to the down-regulation of these genes, which aligned with the findings from transcriptome sequencing. This suggests the possibility that *Rv1476* may inhibit the expression of anti-tuberculosis-related genes, to promote the survival of *M. tuberculosis* in macrophages. 

However, this study has several limitations. The experiment was conducted only with overexpression strains, and the gene was not deleted or supplemented in *M. tuberculosis*. Therefore, further experimental verification is necessary. Additionally, while this study provides a preliminary exploration of the relationship between the biological functions of *Rv1476* and macrophages, the exact mechanism behind this relationship remains unclear. Therefore, further mechanistic research is needed to fully understand this phenomenon.

## 5. Conclusions

In summary, we have preliminarily explored the biological functions of *Rv1476* and its impact on macrophage transcriptome changes. *Rv1476* may affects the expression of macrophage apoptosis and immune response-related genes, thereby promoting the survival of *M. tuberculosis* in macrophages. These findings provide new potential targets for the treatment and prevention of tuberculosis.

## Figures and Tables

**Table 1:** Primer sequences are used in this article.

Primers	Forward Sequence	Reverse Sequence
Rv1476-OE	CATCAGGAGGAATCATCTAGA ATGACCGGGCCATATTTTCCT	GTTAACTACGTCGACATCGAT CTAGACGCCTTGATTGAGATC
Rv1476-qPCR	ACCATTCCAAGACCGGCAAT	CAGTTGCATCAACCGAGCAC
CCL1	TTGGATGGGTTCAGAGGCAC	GGTAGTTTCGGGGACAGGTG
IL15	AGTTGGCCCAAAGCACCTAA	GCACTGAAACAGCTGCACAA
IL16	GCTGGGATGGAAATGCTCCT	TGTTTGTAGGTGACCGCCTC
ISG15	GTTCATGAATCTGCGCCTGC	AGCCTTTATTTCCGGCCCTT
GBP5	CCGCTGCATACAAATCAGGC	ACATGGGGTCTGACATGTGG
IFNB	AGTGTCAGAAGCTCCTGTGG	TAGATGGTCAATGCGGCGTC
ATG2A	GAGATCGCCGGCCAGAAG	CAGGTCACGCTGGTTGATGA
CSF3	CTGGTGAGTGAGTGTGCCA	CCGCTATGGAGTTGGCTCAAG
IL23A	CCAGCTTCATGCCTCCCTAC	TTGAAGCGGAGAAGGAGACG
GAPDH	GACAGTCAGCCGCATCTTCT	GCGCCCAATACGACCAAATC

## Data Availability

RNA-seq data has been deposited in GEO database with accession no. GSE247649.
